# Adhesion of Neurons and Glial Cells with Nanocolumnar TiN Films for Brain-Machine Interfaces

**DOI:** 10.3390/ijms22168588

**Published:** 2021-08-10

**Authors:** Alice Abend, Chelsie Steele, Heinz-Georg Jahnke, Mareike Zink

**Affiliations:** 1 Research Group Biotechnology and Biomedicine, Faculty of Physics and Earth Sciences, Peter Debye Institute for Soft Matter Physics, Leipzig University, Linnéstraße 5, 04103 Leipzig, Germany; cs73gygu@studserv.uni-leipzig.de; 2 Centre for Biotechnology and Biomedicine, Molecular Biological-Biochemical Processing Technology, Leipzig University, Deutscher Platz 5, 04103 Leipzig, Germany; heinz-georg.jahnke@bbz.uni-leipzig.de

**Keywords:** neurons, glial cells, electrode materials, cell adhesion, cell spreading, TiN, nanostructured surfaces, cell-surface interaction, neuroelectrode

## Abstract

Coupling of cells to biomaterials is a prerequisite for most biomedical applications; e.g., neuroelectrodes can only stimulate brain tissue in vivo if the electric signal is transferred to neurons attached to the electrodes’ surface. Besides, cell survival in vitro also depends on the interaction of cells with the underlying substrate materials; in vitro assays such as multielectrode arrays determine cellular behavior by electrical coupling to the adherent cells. In our study, we investigated the interaction of neurons and glial cells with different electrode materials such as TiN and nanocolumnar TiN surfaces in contrast to gold and ITO substrates. Employing single-cell force spectroscopy, we quantified short-term interaction forces between neuron-like cells (SH-SY5Y cells) and glial cells (U-87 MG cells) for the different materials and contact times. Additionally, results were compared to the spreading dynamics of cells for different culture times as a function of the underlying substrate. The adhesion behavior of glial cells was almost independent of the biomaterial and the maximum growth areas were already seen after one day; however, adhesion dynamics of neurons relied on culture material and time. Neurons spread much better on TiN and nanocolumnar TiN and also formed more neurites after three days in culture. Our designed nanocolumnar TiN offers the possibility for building miniaturized microelectrode arrays for impedance spectroscopy without losing detection sensitivity due to a lowered self-impedance of the electrode. Hence, our results show that this biomaterial promotes adhesion and spreading of neurons and glial cells, which are important for many biomedical applications in vitro and in vivo.

## 1. Introduction

Many cellular processes such as proliferation and migration rely on the ability of cells to adhere to a surrounding medium such as the extracellular matrix (ECM) or a biomaterial [1]. In vivo cells mainly connect to the ECM, which constitutes different proteins with specific binding sites for cellular adhesion. In contact with a biomaterial composed of metals or ceramics, these proteins are missing. However, cells express specific proteins, such as fibronectin or laminin, which can then be deposited onto the biomaterial. This enables the cells to form specific adhesion points via surface receptors connecting to the previously deposited proteins. First adhesion sites for cells in contact with biomaterials can occur within seconds when early focal complexes form [2]. Subsequently, maturation of this soft binding step to focal adhesion results in specific adhesion sites in which cellular adhesion receptors interact with ECM molecules and form mature adhesion complexes [2,3].

Within the last decade, a large variety of biomaterials have been developed, ranging from diagnostic tools [4] to dental [5] and orthopedic implants [6], organ replacement [7], and tissue engineering [8] to vascular grafts [9] and pharmaceutical applications [10]. Nevertheless, the functioning of medical devices and implants relies on good integration and cellular adhesion in vivo. In terms of neuroelectrodes used for deep brain stimulation to treat diseases such as Parkinson’s disease [11] or treatment-resistant depression [12], the coupling of neurons to the electrode is crucial for electric signal transfer. However, a mechanical mismatch of the stiff electrode which exhibits Young’s moduli in the range of GPa and the soft brain tissue often generates inflammatory responses and the formation of scar tissue around the electrode, causing degradation of recording and implant failure [13,14]. Additionally, glial scarring hinders regeneration of neurons after local injury, which inevitably has to be accepted after implantation of the electrode [15]. Alternatively, soft [16,17] and bioinspired electrodes [18,19] can overcome this drawback, while implantation strategies are still a matter of debate [20]. Nevertheless, electronic features such as low impedance, and a suitable signal-to-noise ratio have to be ensured. Other strategies employ nanotechnology to reduce scar formation by optimization of neural electrode-tissue interfaces, including carbon nanotube fiber-based surfaces [21] and nano-coatings [21,22].

Brain-machine interfaces such as lab-on-a-chip devices and multielectrode arrays (MEA) also offer great potential for in vitro and in vivo application to study neuronal circuit-connectivity, physiology, and pathology [23]. As recently shown by Vafaiee et al., carbon nanotube modified microelectrode arrays show improved electric properties important for neural interfaces [24]. Currently, 384-multiwell microelectrode arrays are used for the impedimetric monitoring of Tau protein-induced neurodegenerative processes [25]. To further miniaturize future brain-machine interfaces, electric materials with a lowered self-impedance of the electrode are required. A possible candidate is titanium nitride (TiN), which exhibits an increased surface area and allows the shrinking of microelectrode size without losing detection sensitivity [26,27,28,29]. By further increasing the surface area of TiN with a nanocolumnar pattern, we have previously shown that neurons and glial cells cultured on these surfaces exhibit a much better proliferation behavior, in contrast to conventional electrode materials such as gold and indium-tin-oxide (ITO) [30]. In addition to research on multielectrode arrays, the functional properties of primary cortical neurons and neuron-like cells have also been studied on promising organic [31,32,33,34], as well as inorganic [35], memristive brain-machine interfaces.

The formation of glial scars is not present during in vitro application in MEA devices when investigating neurons in a cell culture system. However, adequate coupling of neurons to the underlying MEA is still a major prerequisite in order for neural recording to become possible. Additionally, in terms of neuron-surface interaction, understanding cell adhesion in terms of response and control of cellular interaction with their environment is an important feature during repair mechanisms and possible medical treatment related to diseases of the central and peripheral nervous systems [36]. Thus, promoting neuron adhesion and growth on a biomaterial is an ongoing task.

In our study, we investigated the short-term adhesion behavior of human neuron-like SH-SY5Y and glial-like U-87 MG cells on several electrode materials (TiN, nanocolumnar TiN, ITO, and gold) using atomic force microscopy-based single-cell force spectroscopy. To this end, we analyzed the maximum adhesion force of single cells and measured the total work required to completely detach the cell from the substrate to quantify the bioactivity of the different surfaces. Adhesion on longer time scales goes in line with maturation of specific cell-surface binding sites, e.g., via integrin receptors [37] which correlates with cell spreading and the formation of neurites. Therefore, we further studied the dynamics of spreading and changes in cell growth areas over several days of cultured SH-SY5Y and U-87 MG cells. While the adhesion and spreading behavior of the glial cells was almost independent of the investigated electrode materials, the neurons showed an enhanced cell spreading on TiN and nanocolumnar TiN in contrast to ITO and gold. Additionally, glial cells already developed their maximum growth areas after one day in culture: neurons needed more time to spread, and the final growth areas were observed after 3 days. Together with the observation that the neurons proliferated much better on TiN and nanocolumnar TiN, as well as formed clusters and agglomerations on these surfaces (a hint for improved physiologic behavior as shown before), our study reveals that nanocolumnar TiN exhibits suitable bioactive properties together with enhanced electric features important for brain-machine interfaces.

## 2. Results

**Figure 1:**
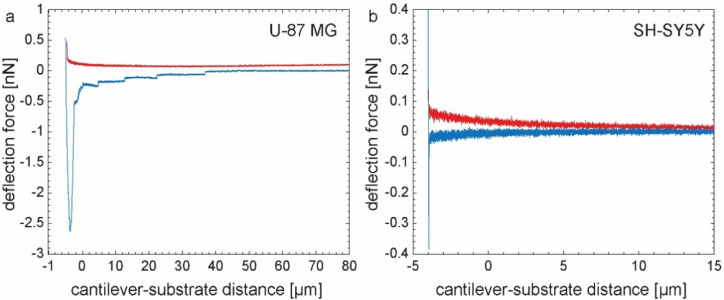
(**a**) Representative example of a force-distance curve of a U-87 MG cell in contact with a TiN substrate. The approach segment is shown in red and the blue graph indicates the retraction of the cell from the sample. The minimum of the retract segment corresponds to the maximum adhesion force; (**b**) Same experiment as in (**a**) but measured with a SH-SY5Y cell.

We investigated the short-term adhesion behavior of U-87 MG and SH-SY5Y cells on several electrode materials (see Figure S1 for information on surface topography, Supplementary Material) using single-cell force spectroscopy for both 5 s and 30 s cell contact times on TiN and nanocolumnar TiN, as well as Au and ITO substrates as control. Representative examples of the resulting force-distance curves are shown in Figure 1. We noticed that characteristic plateau-shaped retract curves mainly occurred for U-87 MG cells (see Figure 1a). The tearing off process while pulling the cell upwards away from the surface happened for the SH-SY5Y cells, usually at much smaller distances from the substrate than for U-87 MG cells. For these cells, we usually observed a single rupture event instead of multiple plateaus as shown in Figure 1b.

**Figure 2:**
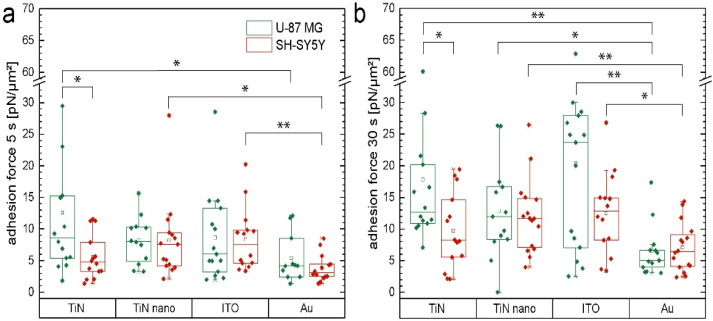
(**a**) Maximal adhesion force of single U-87 MG (green) and SH-SY5Y (red) cells measured with a contact time of 5 s on different electrode materials (TiN, nanocolumnar TiN, ITO, Au); (**b**) Same as in (**a**) but with a contact time of 30 s. We marked data values as significant (*) for *p* ≤ 0.05 and highly significant (**) for *p* ≤ 0.01.

We analyzed the maximum adhesion forces exerted by the cells while being detached from the substrate. The mean values for each cell normalized by the average contact area are shown in Figure 2a,b. We observed that the maximum adhesion values are generally higher for longer contact times for both cell types and all materials. Gold showed the lowest adhesion forces for both neuronal and glial cells with 3.8 pN and 5.4 pN for 5 s contact time, respectively, as well as 7.1 pN and 6.5 pN for 30 s contact time, respectively. The largest adhesion force was found for glial cells on ITO after 30 s with a value of 20.4 pN compared to 8.6 pN for 5 s, while for the neuronal cell type the adhesion force increased from 8.3 pN after 5 s to 12.5 pN after 30 s contact time. TiN and nanocolumnar TiN both delivered a comparable ratio of adhesion forces for U-87 MG and SH-SH5Y cells for different contact times. However, neurons generally comprised comparatively higher adhesion forces on the nanocolumnar TiN surface (12.1 pN for 30 s) with very similar forces as found for the glial cells (12.9 pN for 30 s).

**Figure 3:**
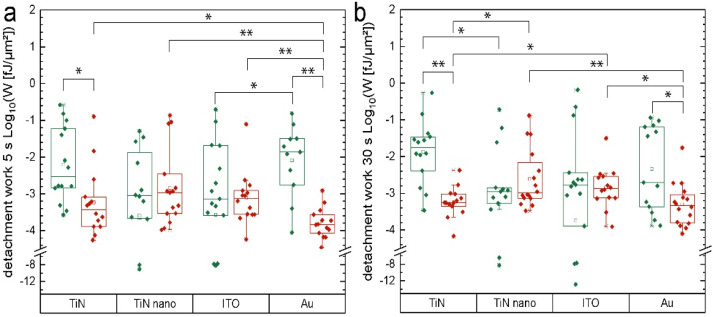
(**a**) Total work required to completely detach a single cell from the electrode substrate with a contact time of 5 s. Results of U-87 MG cells are shown in green and SH-SY5Y cells in red; (**b**) Same as in (**a**) but with a contact time of 30 s. We marked data values as significant (*) for *p* ≤ 0.05 and highly significant (**) for *p* ≤ 0.01.

In addition to the maximal adhesion forces, we also studied the total work required to completely detach a single cell from the electrode substrate, i.e., the area between the retract part of the force-distance curve and the baseline. The mean values for each cell are presented in Figure 3a,b with a logarithmic scale. This detachment work represents the overall cell adhesion because it includes every single separation event while pulling the cell upwards away from the electrode substrate. Interestingly, glial cells generally exhibited a broader distribution of data points with more outliers in comparison to their neuronal counterparts. The electrode material gold shows the lowest median values for SH-SY5Y cells for both adhesion times (with 1.5 × 10^−4^ fJ/µm^2^ for 5 s and 4.7 × 10^−4^ fJ/µm^2^ for 30 s contact time), whereas the U-87 MG cells seemed to adhere much stronger to this substrate type with 1.9 × 10^−3^ fJ/µm^2^ for 30 s and 1.4 × 10^−2^ fJ/µm^2^ for 5 s adhesion time. Comparing the two TiN materials, neurons adhered weaker regarding the detachment work on TiN than nanocolumnar TiN. We see the highest median value of the detachment work on nanocolumnar TiN for SH-SY5Y for 5 s contact time with 1.1 × 10^−3^ fJ/µm^2^. On the other hand, U-87 MG cells exhibited their second-poorest adhesion behavior with a median detachment work of 1.1 × 10^−3^ fJ/µm^2^ on nanocolumnar TiN, right behind ITO with a value of 1.7 × 10^−3^ fJ/µm^2^ for 30 s contact time.

**Figure 4:**
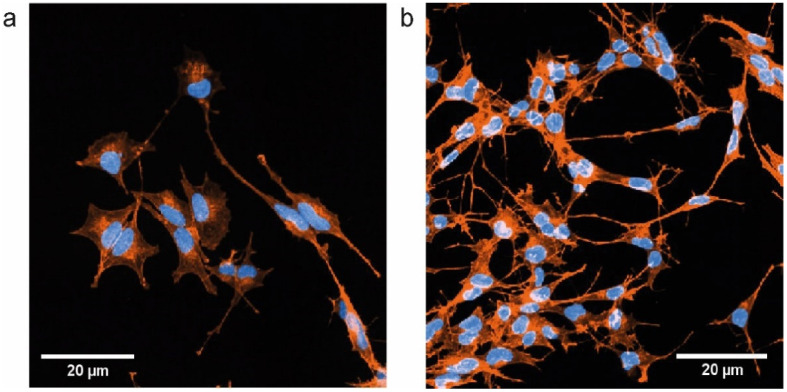
(**a**) Fluorescence image of SH-SY5Y cells grown on ITO substrate for 3 days. Actin fibers are shown in orange and cell nuclei in blue; (**b**) Same as in (**a**) for cells cultured on nanocolumnar TiN.

**Figure 5:**
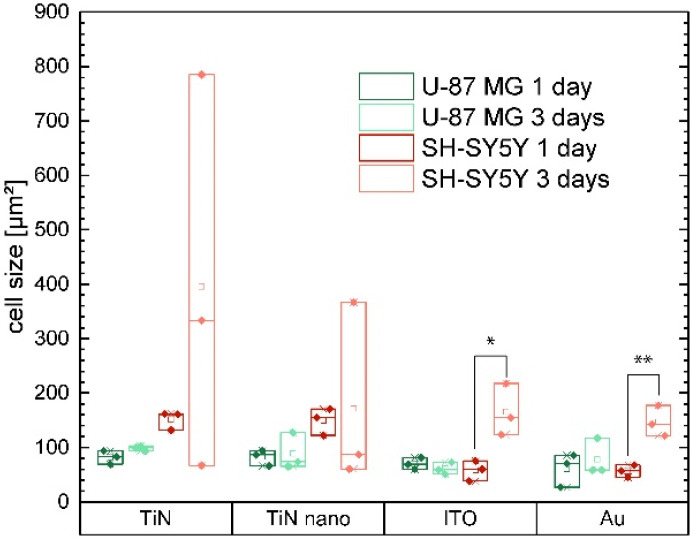
Cell size (viz. projected cell areas) on different substrate materials extracted from fluorescent images of actin phalloidin labeled glial U-87 MG (green) and neuronal SH-SY5Y (red) cells for different growth times. We marked data values as significant (*) for *p* ≤ 0.05 and highly significant (**) for *p* ≤ 0.01.

Additionally, with regards to the analysis of the single-cell short-term adhesion behavior, we also investigated the spreading of cells in networks over longer time scales of several days cultured on the electrode materials. Cell spreading, and thereby increase of cell size, is usually coupled to an increased number of adhesion points, which in turn leads to enhanced cell adhesion and improved bioactivity of the underlying substrate material [38,39]. Here, we measured the size of actin phalloidin labeled U-87 MG and SH-SY5Y cells, viz. the projected cell areas grown on TiN, TiN nano, ITO, and gold electrode substrates for one day and three days, respectively. Examples of the fluorescent images of actin fibers of SH-SY5Y and U-87 MG cells grown on ITO and TiN nano that were used for these experiments are shown in Figure 4 and Figure S2, Supplementary Materials. Results of the projected cell area analysis are presented in Figure 5.

The size of U-87 MG cells after 1 day of culture was almost identical for all substrate materials with values around 90 µm^2^. After 3 days, cell areas hardly increased due to spreading and only small differences became visible. In fact, on ITO, the cell area mean value decreased by around 13% after 3 days compared to 1 day, while on TiN an increase of around 20% was observed.

In contrast, the growth areas of neurons increased strongly from day 1 to day 3 on ITO and Au. We also observed much larger cells on TiN and TiN nano, while on both materials there was great variation of cell sizes after 3 days. Thus, on both materials, cells were found much smaller than seen after 1 day, as well as cell sizes up to 5 times larger. On TiN, the mean value doubled from day 1 to day 3, while on TiN nano, the mean value decreased. Even though very large cells were seen on TiN nano, the number of very small cells was highest. Since the SH-SY5Y cells organized in clusters on the TiN and TiN nano surface in contrast to ITO and Au [30], cells in the center of the clusters could not be included in our evaluation because of the high density, which resulted in optical separation of adjacent cells becoming impossible. Thus, our evaluation only included cells in less dense areas, viz. in the vicinity of cluster centers.

## 3. Discussion

In our study, we investigated the adhesion behavior of both human neuron-like SH-SY5Y and glial-like U-87 MG cells on short time scales of seconds. To this end, single-cell force spectroscopy with cell-material adhesion times of 5 s and 30 s was used to obtain data on the cells’ maximum adhesion force, as well as the work required to completely detach a single cell from the electrode substrate. In our experiments, we do not take wetting behavior effects into account (viz. spreading of cells in contact with a surface due to surface tension effects when cells are considered liquid-like [40]) since the force spectroscopy microscope actively pushes the cell onto the electrode substrate with a constant setpoint of 500 pN, and then sustains the reached z-position for the adhesion time of either 5 s or 30 s, respectively. The results were normalized to the cell-material contact area (see Materials and Methods) to exclude the influence of sheer cell size, which likely correlates with the number of formed adhesion points and therewith adhesion force itself [41]. As expected [42], we found higher values of the cell-substrate adhesion force for both cell lines on all materials for the longer contact time. Our results show the lowest adhesion forces on gold substrates for both neuronal and glial cells. Since bad cell adhesion can also reduce proliferation, our results go in line with our previous findings that neurons and glial cells show a reduced proliferation rate on gold compared to ITO, TiN, and nanocolumnar TiN [30]. However, reduced proliferation rates could also be partly attributed to the influence of differentiative processes on the neuron-like SH-SY5Y cells caused by the surface nanotopography of the substrate material as shown recently [43,44,45]. Optically judging from Figure 4, we did indeed notice differences in the differentiation state of SH-SY5Y cells on particular electrode materials.

Additionally, we noticed an overall broader distribution of adhesion force and detachment work values for U-87 MG cells than SH-SY5Y cells. As previously shown by Dao et al. for CHO cells [46], cell adhesion variability stems from cell to cell variation within populations and does not depend on changes of adhesion behavior of single cells after repetitive measurements and is also likely not due to different cell cycle phases. In contrast, Panagiotakopoulou et al. [47] reported periodic variations of cancer cell traction forces on substrates connected to proliferative cycle phases. Moreover, Lock et al. [48] even identified a specific form of adhesion complex which is assembled during the mitotic phase of cells. The dependency of our cell adhesion force results on cell cycle phase is challenging to analyze because it is difficult to determine the phase of cells which are attached to the AFM cantilever for measurements.

We want to mention that our single-cell force spectroscopy study is subject to similar technical limitations as reported by other research groups, see e.g., Helenius et al. [42]. Specifically, in our case, the adhesion forces exerted by the cells for contact times of more than 30 s were too high to be reliably and reproducibly measurable in our atomic force microscopy setup. Here, adhesion forces originating from contact times above 30 s usually exceeded the measurable range and the cell could not be completely detached from the substrate, which leads to our decision to limit the single-cell force spectroscopy measurements to contact time values up to 30 s. We attribute the increase in binding strength to the beginning of specific binding site formations. Dot-like nascent adhesions and focal complexes form on time scales of tens of seconds before maturation into focal adhesions occurs, matching the time scales employed in our single-cell force spectroscopy measurements [2,49,50,51,52,53].

As recently shown by Chighizola et al. for SCFS measurements of PC12 cells, adhesion forces of these cells in contact with nanostructured colloidal ZrO_2_ probes revealed a reduced number of adhesion sites after 60 s contact time [54]. This drop is considered to be caused by mechanotransductive interactions at the cellular level which result in excessive force loading in single adhesion sites. This process was not observed on the respective time scales when flat ZrO_2_ probes were employed. In our experimentally accessible time scales, such force drop as a possible early marker of integrin adhesion complex maturation was not visible. However, since the size of focal adhesions depends on their maturation stage [55,56], we expect that for differently structured nanosurfaces with various length scales, time scales on which the observed actomyosin-generated forces occur might vary as well.

Due to the short time scale of our experiments, it is challenging to conclude which type of substrate material supports maturation of focal contact adhesions best, which is important for long-term cell behavior. In fact, interaction of cells and substrate materials can have a great variety of short-term, as well as long-term, consequences on cell behavior and functions, such as adhesion complex formation, spreading, proliferation, differentiation, gene expression, and mechanotransduction. Unraveling the connections between these diverse interactions remains challenging and even the definition of “good adhesion” can vary greatly for different cell types. In case of neurons, delicate cellular adhesion is not automatically a disadvantage [57,58]. Thus, in order to better understand adhesion of neurons and glial cells on the different electrode materials, we compared the single-cell force-spectroscopy data with long-term adhesion studies in order to investigate possible correlations between short-term adhesion and the spreading area of cells as an indicator of strong cell-surface interaction. To this end, we calculated the growth areas of cultured cells after one and three days on the four different materials. However, it is important to note that cellular organization and proliferation can strongly influence spreading: fast proliferation and the formation of cell clusters lead to a reduction of substrate space available for spreading.

As we have shown previously, U-87 MG cells showed the poorest cell proliferation on gold substrates (the cell number increased on gold by a factor of 1.6 and nearly tripled on ITO, TiN, and nanocolumnar TiN with additional growth time) [30]. Thus, the available space for spreading was largest on gold. However, the size of their cell bodies did not increase significantly. Hence, the adhesion behavior of glial cells hardly evolves on gold substrates over time. In contrast, U-87 MG cells proliferated much better on ITO, TiN, and nanocolumnar TiN substrates but still lacked a significant increase in cell size. The cells tended to form evenly distributed cell patterns instead of agglomerations and clusters on these materials [30] which would allow spreading in the empty intercellular spaces. However, we saw no significant changes in cell area, and thereby, cell adhesion for the glial cells. Overall, U-87 MG tended to behave the same on all utilized materials: cells arranged themselves in homogenous agglomeration-free patterns and hesitated to change their size independent of their proliferation behavior.

In contrast, neuronal SH-SY5Y cells exhibited a completely different response in contact with the electrode materials. We noticed an increase in cell size on gold and ITO, but the number of cells was halved on gold and stagnated on ITO after three days of growth time in comparison to the one-day experiment. The cells were distributed homogeneously and had enough space to spread (for an example of cell distribution on a surface see Figure S3, Supplementary Material). Additionally, optical inspection of cells revealed that cell networks lacked alterations: there were no new neurite formations, which in turn lead to only slight changes of the overall cell size since the central cell body hardly changed in size. We noticed only small variances of the cell size data of neuronal cells on TiN and TiN nano after one day. The cells had not yet formed clusters on the substrates and all cells had more or less the same amount of space available to them for spreading. The situation changed after additional growth time when the number of SH-SY5Y cells had more than doubled, and the variance of cell sizes increased significantly. There were now giant and tiny cells that were only half as big as the mean cell size after one day. The cells started to agglomerate in clusters, whereas the cluster density was larger on TiN nano than TiN (for an example of cluster formation see Figure S4, Supplementary Material). This could very well give answers to the question of why there were overall fewer giant cells visible. Even if areas of very high densities are excluded from the results due to technical properties of the analysis algorithm which only allowed us to quantify cells we could fully see and distinguish from their immediate neighbors, the SH-SY5Y cells still grew densely and appeared therefore on average smaller than on TiN. There were areas in the vicinity of cell clusters and in the inter-cluster space of the samples where cells grew very large. This means that the cells produced long neurites and formed networks in the less-occupied substrate areas to connect with the cells in clusters. In summary, SH-SY5Y cells tended to spread more on TiN and nanocolumnar TiN in areas where there was enough empty space available in comparison to the ITO and gold materials. This suggests an increased adhesion of neuronal cells on TiN and TiN nano. Moreover, the cells formed more and longer neurites on these materials to build a network with cells in high-density areas in clusters (see Figure 4 and Supplementary Material). This behavior mirrors physiological conditions much better than a homogeneous cell distribution and weaker network formation of SH-SY5Y cells on ITO and gold.

We have to emphasize that during short-term adhesion, as present during single-cell force spectroscopy, unspecific bonds formed during cell-surface contact, while for longer culture times specific bonds, e.g., via integrin binding motifs, occurred [59]. Formation of such specific ligand-receptor pairs is also the origin of the observed neurite formation [60] mainly seen on TiN and TiN nano. Since TiN and TiN nano promote cell spreading to a much larger extent compared to gold and ITO (in fact the size of the cell body remained almost constant and it was the formation of new neurites which contributed to the growth area increase), we conclude that the maturation of specific bonds is directly correlated with cell size during long-term adhesion and a measure for the surfaces’ bioactivity.

The effect of nanostructured surfaces on adhesion and proliferation of SH-SY5Y was recently reported by Boehler et al. [61]. They showed that nanostructured platinum coatings of neuroelectrodes, as well as unstructured surfaces, do not exhibit cytotoxic effects on the SH-SY5Y cells. Dominguez-Bajo et al. [62] grew neuronal cells derived from rats on both flat and nanostructured nickel and gold electrodes. Increased neural cell survival, improved neuronal differentiation, and fewer glial cells were measurable on nanostructured nickel in comparison to its flat counterpart. The surface topography of the gold samples seemed to have only little effect on cell proliferation and differentiation, while the nanostructure still reduced the number of glial cells in culture. Thus, apparently, the electrode material’s surface chemistry, as well as topography, seems to play a major role in finding biocompatible candidates. In our experiments, we do not attribute differences in cell adhesion on Au and TiN substrates to surface roughness differences, since both materials exhibited a very similar RMS roughness and similar grain sizes [30]. Nevertheless, adhesion and spreading of neurons were different on these materials, most likely due to chemical influences.

In terms of tissue adhesion in contrast to single-cell adhesion, we want to point out that our previous study with neuronal tissues such as adult retinae and adult brain slices in contact with TiO_2_ nanotube scaffolds clearly showed that the size of the nanotubes, viz. the length scale of the surface nanostructure, strongly influences tissue adhesion and cell viability [63,64]. From this study, we expect that the nanostructure of an electrode material might promote adhesion of neurons and even reduce the risk of glial scar formation and encapsulation, an important feature for the application of a neuroelectrode in vivo. Thus, even though the mechanical mismatch between brain tissue and electrode material cannot be avoided, the nanotopography of the electrode’s surface might be a valuable tool to improve the connection between neurons and electrode. To this end, future studies should address the interaction of neuronal tissue explants with nanocolumnar TiN to determine tissue adhesion ex vivo, while in vivo investigation of the material can give rise to the question if a nanostructure can reduce glial scarring.

Finally, we want to mention that the bioactivity of a material is often connected to the ability of protein adsorption, which in turn is important for cell adhesion. As we have shown previously, surface topography can change the adsorption behavior, and surfaces that promote fibronectin adsorption exhibit an enhanced ability for improved cell adhesion and spreading [39,65]. To investigate possible differences in protein adsorption behavior, we soaked our Au, ITO, TiN, and TiN nano materials in purified water including fluorescently labeled laminin, an extracellular matrix protein, which is widely expressed in the central nervous system and important for specific binding of the ECM to neurons (see Figure S5, Supplementary Material). In fact, we observed only very small differences in laminin adsorption. Thus, we conclude that the observed cell behavior in terms of short-term adhesion and spreading after 1 to 3 days is hardly influenced by possible protein adsorption, but determined by other material properties such as chemical cues.

## 4. Materials and Methods

### 4.1. Electrode Materials Preparation

The following substrates were used for our experiments: indium tin oxide (ITO), gold (Au), as well as titanium nitride in two different surface topographies (TiN and TiN nano). All materials were produced by thin film deposition on glass cover slits and characterized in terms of surface topography by electron microscopy and atomic force microscopy as described previously in Abend et al. [30]. Briefly, the different films exhibit the following features: the gold substrates showed the lowest root-mean-square (RMS) roughness and even transitions between the individual grains. In contrast, ITO exhibited the highest RMS roughness of all four materials and a crystalline surface structure. TiN samples were produced with two different sputter times which lead to a film thickness of 150–200 nm for TiN and 500–550 nm for TiN nano and widely different surface morphologies. TiN showed a cauliflower-like formation with several grain sizes, whereas TiN nano exhibited a nanocolumnar structure with single-type grains. This resulted in a surface area increase of (1.27 ± 0.08) nm for TiN nano. All of the other materials scored below 1.1 with (1.02 ± 0.01) nm for Au, (1.10 ± 0.02) nm for ITO, and (1.07 ± 0.01) nm for TiN (for more details see Abend et al. [30]).

### 4.2. Cell Lines and Cell Culture for Single-Cell Force Spectroscopy

We used the glioblastoma cell line U-87 MG (Cat.No. 300367, CLS Cell Lines Service GmbH, Eppelheim, Germany) and the neuroblastoma cell line SH-SY5Y (Cat.No. CRL-2266, ATCC LGC Standards GmbH, Wesel, Germany) for our studies. Both cell lines were grown in culture flasks in a 1:1 mixture of MEM Eagle/Ham’s F12 medium with Earle’s salts, L-glutamine, and sodium bicarbonate (Cat.No. M4655 and N6658, Sigma-Aldrich Chemie GmbH, Munich, Germany) and kept at 37 °C in a 95% air and 5% CO_2_ atmosphere. Medium change was performed every 2–3 days. We supplemented the cell culture medium with 10% fetal bovine serum (Cat.No. S0615, Biochrom GmbH, Berlin, Germany) and 1% penicillin/streptomycin (Cat.No. P0781, Sigma-Aldrich Chemie GmbH, Munich, Germany). Cells were passaged using phosphate-buffered saline (PBS, Cat.No. 18912014, Gibco, Thermo Fisher Scientific, Waltham, MA, USA) with 0.025% (*w*/*v*) trypsin and 0.011% (*w*/*v*) ethylenediaminetetraacetic acid (EDTA, Cat.No. L2143, Biochrom GmbH, Berlin, Germany). Trypsinization took 3–4 min for each culture flask.

For normal cell passages, regular serum-containing cell culture medium was added to the detached cells to inactivate the trypsin, and cells were seeded in fresh complete medium afterward. To perform single-cell force spectroscopy experiments with the detached cells, serum-free medium consisting of a 1:1 mixture of MEM Eagle/Ham’s F12 medium containing Earle’s salts, L-glutamine, and sodium bicarbonate (Cat.No. M4655 and N6658, Sigma-Aldrich Chemie GmbH, Munich, Germany) supplemented with 1% penicillin/streptomycin (Cat.No. P0781, Sigma-Aldrich Chemie GmbH, Munich, Germany) was added to the detached cells. Cells were then centrifuged for 1 min at 800 rounds/min. The resulting supernatant liquid was aspirated and the cell pellet was resuspended in fresh serum-free cell culture medium.

### 4.3. Single-Cell Force Spectroscopy

We quantified adhesion of glial cells (U-87 MG) and undifferentiated neuron-like cells (SH-SY5Y) on electrode materials (Au, ITO, TiN, and TiN nano) by single-cell force spectroscopy. Prior to measurement, we attached a single cell to a tipless arrow-shaped cantilever (Arrow™ TL1, NanoWorld AG, Neuchâtel, Switzerland) utilizing a Poly-D-Lysine coating (PDL, Cat.No. A-003-M, Sigma-Aldrich Chemie GmbH, Munich, Germany). PDL was diluted with sterile PBS to a concentration of 20 µg/mL and each cantilever was coated with 50 µL of that solution in a petri dish overnight at 4 °C. Each cantilever was used once and cleaned after a measurement with one cell using piranha etch solution consisting of 70% sulfuric acid (Cat.No. 84727, Sigma-Aldrich Chemie GmbH, Munich, Germany) and 30% hydrogen peroxide (Cat.No. H1009, Sigma-Aldrich Chemie GmbH, Munich, Germany).

For single-cell force spectroscopy, the CellHesion atomic force microscope (CellHesion 200, JPK BioAFM–Bruker Nano GmbH, Berlin, Germany) equipped with the JPK Instruments SPM and DP software Version 6.1.146 was used for the cell adhesion experiments. A CCD camera (FireWire CCD Color Camera DFK 41AF02, The Imaging Source Europe GmbH, Bremen, Germany) was mounted onto the CellHesion system to visualize cell capture and adhesion to the cantilever. For our experiments, we used standard 4 cm petri dishes (TPP Techno Plastic Products AG, Trasadingen, Switzerland) coated with a solution of 1% bovine serum albumin (BSA, Cat.No. A2153, Sigma-Aldrich Chemie GmbH, Munich, Germany) diluted in PBS. The dishes were stored at 4 °C overnight, shortly rinsed with Millipore water, and dried with nitrogen the next day. An electrode substrate was glued to the petri dish bottom using nail polish to avoid slippage. Once the polish had dried completely, 2 mL serum-free cell culture medium was added and the dish heating system was set to 37 °C. Additionally, an atmosphere consisting of 95% air and 5% CO_2_ was created. A PDL-coated cantilever was then mounted on the glass block of the CellHesion-AFM. After reaching thermal equilibrium in the 37 °C warm serum-free culture medium, the cantilever was then calibrated with the built-in contact-based calibration tool of the JPK software, which is based on the spring constant calibration method proposed by Hutter and Bechhoefer [66]. Cells were passaged as described in the section above. The scanning head of the CellHesion-AFM was briefly removed to flush 10 µL of the cell solution into the petri dish. One cell of average size and smooth spherical form was chosen for the measurement. It was captured by positioning the tip of the cantilever over the cell and running a single scan repetition in the constant force mode with a 500 pN setpoint, 5 µm/s constant speed, 5 s contact time and 100 µm pulling length. Once the cell was successfully captured, we waited 30 min before starting the measurements to ensure that the cell firmly adhered to the cantilever. The cantilever was then positioned over the electrode substrate in the petri dish and the cell adhesion behavior on the material was probed by acquiring force-distance curves with 500 pN setpoint and 100 µm pulling length. The extend speed was set to 2 µm/s and retract speed was chosen as 1 µm/s for each scan repetition. Contact time was set to either 5 s or 30 s. The cell was probed 5 times with an adhesion time of 5 s with 120 s recovery breaks in between repetitions. After a 5 min rest time, the cell was probed again 5 times with a contact time of 30 s and 120 s recovery breaks in between, respectively. The position of the cell on the electrode substrate was changed after every run. Having finished the measurements with one cell, the cell culture medium in the petri dish was replaced with fresh serum-free medium. The cantilever was replaced by a new one with a different cell attached to repeat the entire experimental procedure.

### 4.4. Growth Area of Cells

The growth area of both U-87 MG and differentiated SH-SY5Y cells on electrode materials (Au, ITO, TiN, and TiN nano) was determined using fluorescence microscopy. We employed the following protocol as previously described [30]. Briefly, for this experiment, we seeded the cells at a density of 130 cells/mm^2^ onto electrode substrate materials (Au, ITO, TiN, TiN nano). Accurate cell numbers were obtained using an automatic optical cell counter (EVETM, NanoEntek Inc., Seoul, Korea). The U-87 MG cells were fixed with paraformaldehyde (Cat.No. HT5011, Sigma-Aldrich Chemie GmbH, Munich, Germany) for 15 min. at time points of 24 h or 72 h after seeding. We did not utilize longer culture times because usually cells then start to grow into dense layers which hinders the cell area analysis. After fixation, cells were washed with PBS and treated with 1% (*w*/*v*) Triton X-100 (Cat.No. 9002-93-1, Sigma-Aldrich Chemie GmbH, Munich, Germany) and 0.5% (*w*/*v*) bovine serum albumin (Cat.No. A2153, Sigma-Aldrich Chemie GmbH, Munich, Germany) for 10 min at room temperature as preparation for fluorescent labeling of actin fibers and cell nuclei. To this end, 1 µg/mL Hoechst 34580 (Cat.No. H21486, Molecular Probes, Eugene, OR, USA) and 0.44 µM Alexa Fluor 532 Phalloidin (Cat.No. A-22282, Molecular Probes, Eugene, OR, USA) diluted in PBS was added to the cells at room temperature for 15 min. Cells were washed again with PBS and placed upside down in petri dishes (Cat.No. 80136, ibidi GmbH, Gräfeling, Germany). We applied mounting medium (Cat.No. 50001, ibidi GmbH, Gräfeling, Germany) between the sample and the petri dish. Specimens were stored at 4 °C before imaging.

Treatment of samples with SH-SY5Y instead of U-87 MG cells was slightly different: we added 25 nM staurosporine (STS, Cat.No. S5921, Sigma-Aldrich Chemie GmbH, Munich, Germany) to the SH-SY5Y samples 24 h after seeding to initiate the cell differentiation process, which takes 72 h to complete [59]. Half of the SH-SY5Y specimens were fixed directly upon removing the STS. For the remaining samples, we replaced the STS containing medium with regular growth medium and let the cells grow for another 72 h before fixation. Subsequently, cells were fluorescently labeled as reported before. The cell network morphology was imaged using confocal laser scanning microscopy. We employed an inverted Zeiss Axio Observer.Z1 microscope equipped with a spinning disk unit (Yokogawa CSU-X1A 5000, Tokyo, Japan) for image acquisition. An array of dual-channel images of whole cell networks was taken with a 25 × glycerin immersion objective for each sample. Each file enclosed a substrate area of 0.22 cm^2^. Up to 54 individual images were required to cover the complete sample area.

The images of actin fibers were used to determine the cell growth area. We processed them with a Fiji distribution [67] (Windows 10, 64-bit version) based on ImageJ software [39,68]. Images were thresholded, binarized, and subsequently analyzed with the edge detection of the particle tracker tool to detect cell shapes and determine the size of the cells. The images of cell nuclei were processed similarly and used for cell counting. Results of the cell proliferation analysis can be found in our previous publication [30].

### 4.5. Statistical Analysis

We employed CellHesion single-cell force spectroscopy measurements to determine the adhesion force of 15 cells for each of the 4 substrate types and 2 adhesion times (5 s and 30 s). For each cell, 10 force-distance curves were acquired, i.e., 5 recordings for each of the adhesion times. Overall, 120 cells were used for the single-cell spectroscopy experiments which resulted in the acquisition of 1200 force-distance curves. The JPK Data Processing software (JPK Data Processing Version 6.1.169, JPK BioAFM–Bruker Nano GmbH, Berlin, Germany) was used to extract the maximum adhesion force and the work required to completely detach a single cell from the electrode substrate of each force-distance curve.

We repeated the cell area measurements three times for each cell type and material combination. In total, we analyzed 160,000 cells.

Two-sample *t*-test in OriginLab software (OriginPro 2017G, OriginLab Corporation, Northampton, MA, USA) for unequal sample sizes was employed to analyze statistical significance of data sets. We marked data values as significant (*) for *p* ≤ 0.05 and highly significant (**) for *p* ≤ 0.01.

## 5. Conclusions

Many biomedical applications such as neuroelectrodes, for example, rely on fine-tuned coupling of cells and tissue to the electrode’s surface. Thus, cell survival, proliferation, and biochemical function all depend on the electrode materials’ surface chemistry and topography. We investigated the short-term adhesion dynamics and long-term evolution of cell spreading and neurite formation with culture time of human neuron-like SH-SY5Y and glial-like U-87 MG cells on four different electrode materials (TiN, TiN nano, ITO, and gold). We found the adhesion behavior of U-87 MG cells to be mostly independent of the substrate material and we found cells stopped spreading after one day of culture time. In contrast, neuronal cells spread much better on TiN and nanocolumnar TiN in comparison with ITO and gold. Here, spreading was mainly determined by the formation of long and numerous neurites for longer culture times. The lowered self-impedance of nanocolumnar TiN combined with our findings of the cells’ adhesion and spreading makes the material a promising candidate for building miniaturized microelectrodes. Even though our material might induce the formation of glial scars due to the mechanical mismatch of the material and the brain tissue, novel treatments are under consideration to restore local neuron density and improve the long-term recording stability as recently shown by Zhang et al. [69]. Moreover, Shur et al. recently demonstrated that soft printable coatings improve the mechanical properties on the electrode surface towards more physiologic conditions important to reduce the formation of scar tissue [70]. Thus, surface structures that promote coupling to neurons, as seen in our study, offer great perspectives for brain-machine interfaces, ranging from MEA to deep brain stimulation. Future studies should address the interplay of neurons and glial cells on nanocolumnar TiN, e.g., in a co-culture system of primary cells or even in an in vivo system to further develop our nanostructured TiN towards possible biomedical applications. Here coatings with suitable electric properties might be a valuable option to enhance neuron adhesion important for long-term recording in vivo.

## Supplementary Materials

The following are available online at https://www.mdpi.com/article/10.3390/ijms22168588/s1.
