# Supplementary material for: Adhesion of Neurons and Glial Cells with Nanocolumnar TiN Films for Brain-Machine Interfaces
Source: Int J Mol Sci. 2021 Aug 10;22(16):8588. doi: 10.3390/ijms22168588 (PMC8395253; doi:10.3390/ijms22168588)
Supplement: Supplementary file 1 [file ijms-22-08588-s001.zip › ijms-1287693-supplementary.pdf]

International Journal of
Molecular Sciences

Supplementary Material

Adhesion of neurons and glial cells with nanocolumnar TiN films for brain-machine interfaces

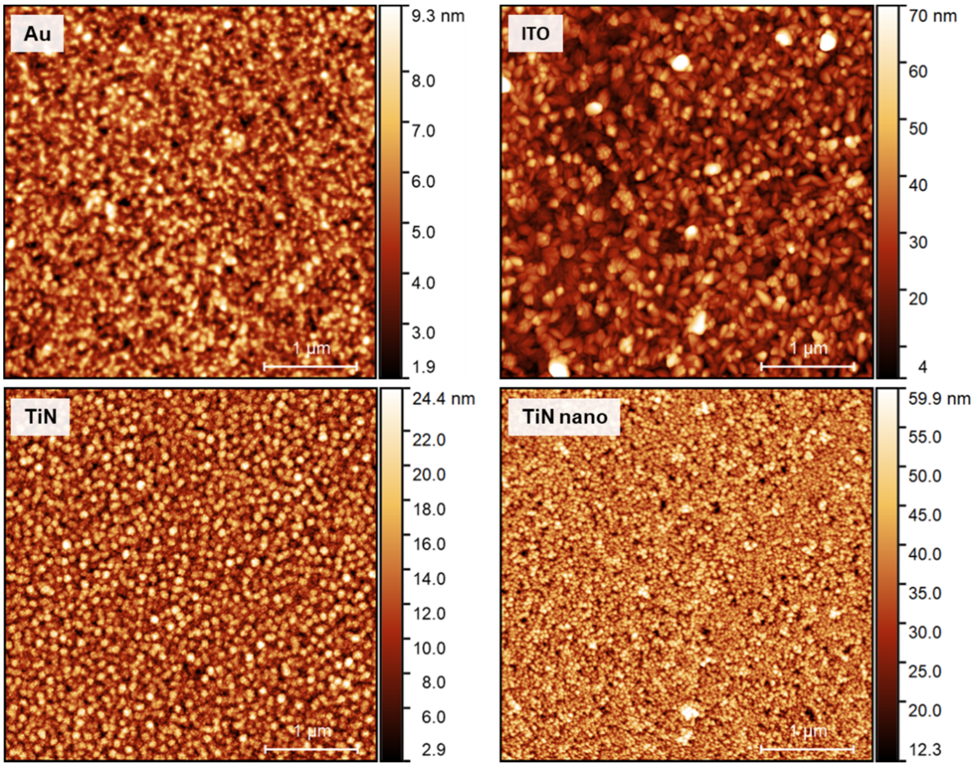

Figure S1. Atomic force microscopy-based topology characterization. The electrode materials of gold (Au), indium tin oxide (ITO), titanium nitride (TiN), and titanium nitride with nanocolumnar structure (TiN nano) were imaged with a JPK NanoWizard 3 atomic force microscope in direct drive AC mode with a TESPAHAR cantilever (Bruker).

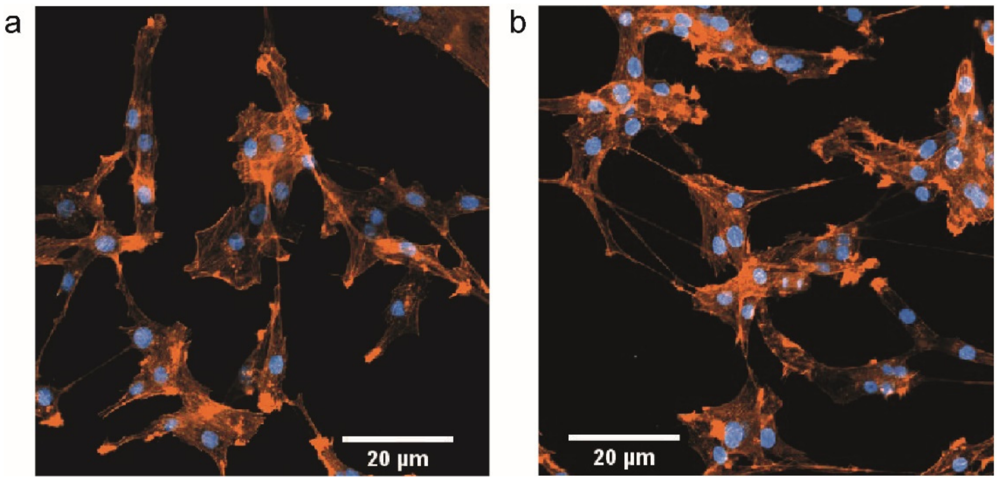

Figure S2. (a) Fluorescence image of U-87 MG cells grown on ITO substrate for 3 days. Actin fibers are shown in orange and cell nuclei in blue; **(b)** Same as in **(a)** for cells cultured on nanocolumnar TiN.

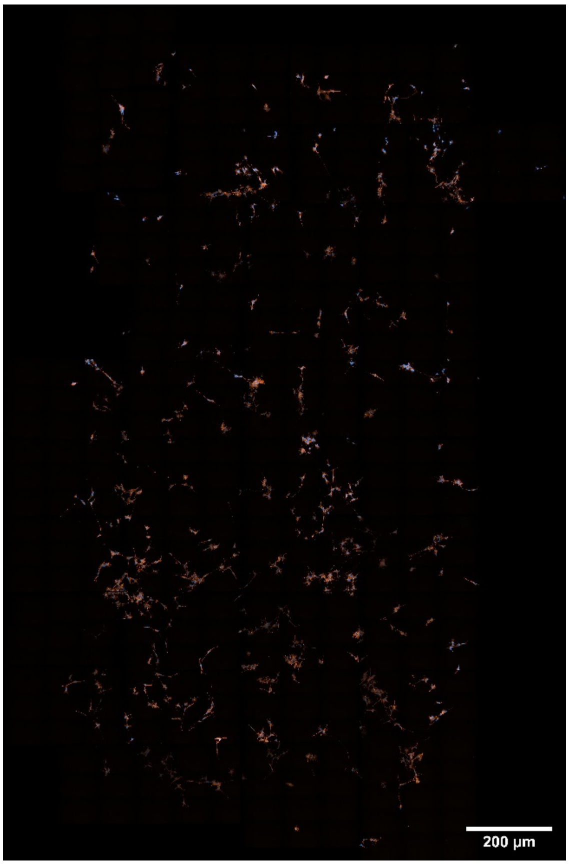

Figure S3. Fluorescent image of SH-SY5Y cells grown on ITO substrate for 3 days after differentiation. Actin fibers are shown in orange and cell nuclei in blue.

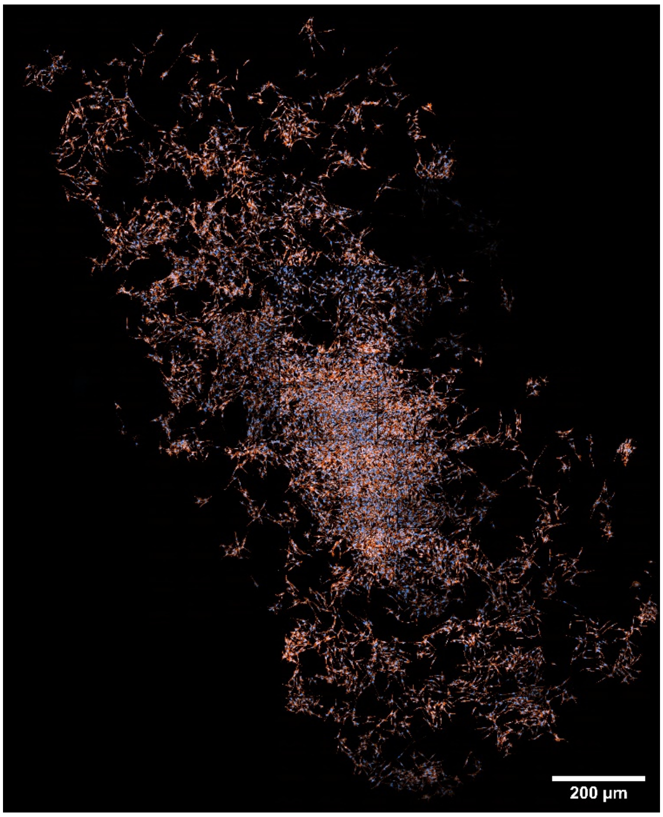

Figure S4. Fluorescent image of SH-SY5Y cells grown on nanocolumnar TiN substrate for 3 days after differentiation. Actin fibers are shown in orange and cell nuclei in blue.

Laminin adsorption

We quantified the adsorption of the cell matrix glycoprotein laminin on electrode materials (Au, ITO, TiN, and TiN nano) using microplate reader measurements. To this end, we applied rhodamine-labeled laminin (Cat.No. LMN01, Cytoskeleton Inc., Denver, Colorado, USA) with a concentration of 10 µg/ml diluted in Millipore water to an area of 0.22 cm^2 resulting in a final concentration of 1.5 µg/cm^2 to the electrode substrates – a coating concentration often used for cell culture, see e.g. Pixley et al. [1] – and incubated the samples for 30 min at room temperature protected from light. Afterwards, non-adsorbed liquid was pipetted into a 96-well plate (Cat.No. 655086, Greiner Bio-One GmbH, Frickenhausen, Germany) and the fluorescence signal was analyzed with a microplate reader (Synergy H1 microplate reader, BioTek Instruments GmbH, Bad Friedrichshall, Germany). The light units were converted into protein mass using a calibration curve ranging from 0 µg to 1.5 µg rhodamin laminin diluted in Millipore water. We repeated this experiment three times for each electrode material type. The results of the analysis are shown in Figure S3. The relative adsorption is the difference between the initially applied and the non-adsorbed protein mass divided by the initial protein mass.

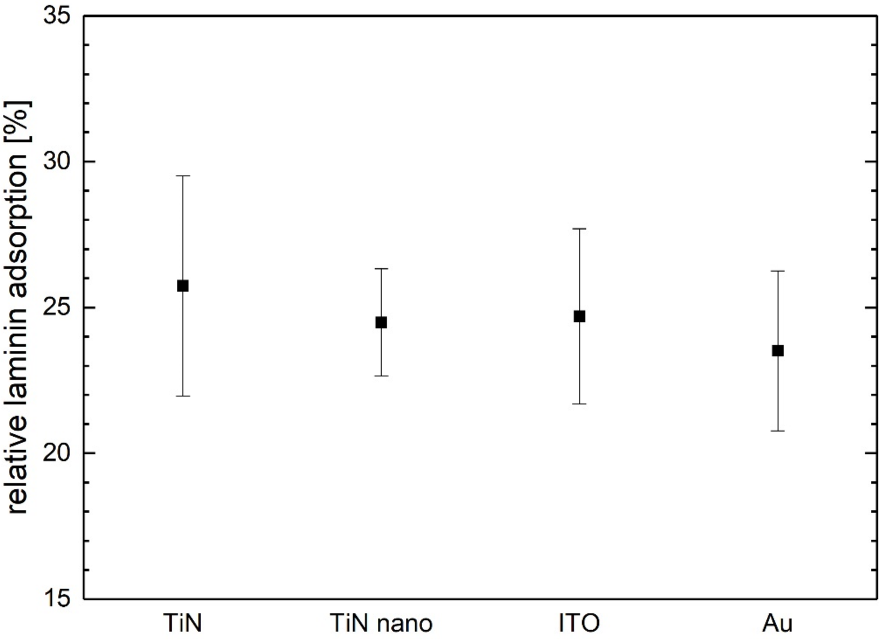

Figure S5. Relative laminin adsorption on different electrode material substrates.

References

1. Pixley, S.K.R.; Nieto-Sampedro, M.; Cotman, C.W. Preferential adhesion of brain astrocytes to laminin and central neurites to astrocytes. *J. Neurosci. Res.* **1987**, *18*, 402–406, doi:10.1002/jnr.490180304.
